# The effect of the Baby-Feed website intervention on diet quality in healthy infants: a randomized controlled trial

**DOI:** 10.1186/s12966-026-01934-9

**Published:** 2026-05-30

**Authors:** Alayne Gatto, Madison Paes-Leme, Sophia Acosta, Mariana Leon, Gabriella Reingevurts, Catherine Coccia, Cristina Palacios

**Affiliations:** Department of Dietetics and Nutrition, Robert Stempel College of Public Health & Social Work, Florida International University, 11200 SW 8th Street AHC5, Miami, FL 33199 USA

**Keywords:** Infant, Caregivers, Diet quality, Website intervention

## Abstract

**Background:**

Current infant diet recommendations are often not followed by caregivers, impacting their infant's diet quality. Incorporating diet guidelines with real-time feedback via technology may improve infant diet quality, helping mitigate future obesity risk. This study reports the effectiveness of the Baby-Feed website in improving diet quality among US infant caregivers compared to controls. This website was designed using trusted behavior-change theories to provide real-time feedback on infant diets, show current infant diet recommendations, and monitor progress.

**Methods:**

Primary infant caregivers (English or Spanish-literate) with internet access, cellphone text capability, and willing to participate in the full study were enrolled into a parallel-group, randomized controlled trial. Infant diet quality was assessed by the total Diet Quality Index Score (DQIS) at baseline (~ 4 months old) and at the end of the study (~ 9 months old). The DQIS (0–55) is scored as: for 0–5.9 months, 15 points for exclusive and 10 points for partial breastfeeding, and 5 points for formula, while other foods score 5 if not introduced and 0 if introduced; for 6–12 months, appropriate intake of each food group (including formula) scores 5 (2.5 if slightly off, 0 if outside recommendations), with same score for breastfeeding. Total DQIS was categorized as ≥45 (excellent) or below. Data were analyzed with ANCOVA or logistic regression, adjusting for infant age and energy intake.

**Results:**

One hundred fifty-five participants were randomized, and 150 were included in the analysis (76 in the intervention group and 74 in the control group). At the end of the intervention, infant total DQIS was higher in the intervention group (44.6 ± 6.53) than in the control group (42.5 ± 7.5) in both the unadjusted (*p* = 0.036) and adjusted models (*p* = 0.046). Also, infants in the control group had twice the odds of having an infant total DQIS below excellent (adjusted OR = 2.00; 95% CI: 1.012–3.950; *p* = 0.046) compared to the intervention group.

**Conclusions:**

Using the Baby-Feed website for 6 months improved the quality of infant diets. This could be a tool to help caregivers follow current infant diet recommendations with real-time feedback.

**Trial registration:**

Clinicaltrials.gov Identifier: NCT05990439.

**Supplementary Information:**

The online version contains supplementary material available at 10.1186/s12966-026-01934-9.

## Background

Establishing healthy behaviors early in life helps prevent obesity, which is one of the premises for nutritional guidance [1, 2]. It is well known that certain nutritional risk factors, such as early breastfeeding cessation, early introduction of complementary foods and beverages (CFBs), introduction of juice or sugar-sweetened beverages (SSBs)/non-core beverages, and poor diet quality, may all contribute to obesity risk in infancy and later in life [3–5]. Therefore, health organizations have provided recommendations and guidelines on infant diets to United States (US) healthcare providers via resources such as websites, guides, and books, to then educate their patients [3–6]. However, these guidelines are often not followed for a myriad of reasons, including structural bias, cultural differences, lack of lactation support, and limited maternity leave [3–5]. For example, breastfeeding rates are significantly below recommendations for exclusive breastmilk for 6 months or for 2 years in the US [5], with only 55.8% of infants receiving breastmilk at 6 months and only 35.9% at 1 year [7]. Parents in the US often introduce CFBs before the 6-month recommendation [8, 9], with 51% introducing them before 6 months [9]. Also, about 24% of parents give infants 100% fruit juice in their first year in the US [10], which is not recommended during that time.

Several interventions have been implemented at well-baby/well-child visits and home visits to educate caregivers on diet guidelines [11–18]. Some have shown improvements in obesity risk with improved nutrition behaviors, such as increasing fruit and vegetable consumption and avoiding SSBs. The interventions to provide nutrition guidance during well-baby visits may be limited by appointment time constraints and by the lack of nutrition training among healthcare providers [19]. Therefore, designing a technology-based intervention may be more appropriate for today’s caregivers, who often seek information online and rely on easily accessible content from social media influencers, blogs, and parenting groups. However, relying on unverified online sources may increase the risk of misinformation [20–23]. And, although infant and pediatric registered dietitians are the nutrition experts, they are not a routine referral in infant well-baby care [24].

Delivering the nutritional education intervention using technology includes advantages for caregivers, such as eliminating transportation challenges. Additionally, web-based options with engaging personalized materials and real-time feedback can help reinforce strategies [25–27]. Some of these interventions have been successful. For example, The Early Food for Future study asked Norwegian caregivers to access a webpage with monthly short video clips on complementary foods and other feeding topics. The intervention led to increased fruit and vegetable consumption among infants [28]. The Grow2Gether study used private Facebook groups facilitated by a psychologist, providing a curriculum of weekly videos on parenting topics, including feeding, which led to significantly higher healthy feeding behavior scores [29, 30].

Among available technology-based interventions, only a few have incorporated a theory of behavioral change in their development [31, 32]. Interventions grounded in theory may be more effective at changing behavior than those without theory [33]. Also, none have included real-time feedback to help caregivers quickly, easily, and accurately improve the infant’s diet. Lastly, most interventions have not evaluated sex differences when evaluating infant diet quality, despite growth charts developed by sex. Although a 2016 systematic review did not find differences in parental control by sex, most studies were among older children [34], and diet quality was not evaluated. Therefore, it is unknown if there are sex differences in diet quality early in life.

Therefore, to fill these gaps, we designed the Baby-Feed website, grounded in two behavior-change theories [35, 36]. The Social Cognitive Theory (SCT) [37] supports interventions by incorporating environmental influences that surround an individual as well as and interpersonal factors, to facilitate a desired change in their behaviors and, the Health Self-Empowerment Theory (HSET), which emerges from a recognition of personal variables, such as health self-efficacy and health motivation and can predict an individual’s purposeful engagement in attainment of health goals [38]. This website provides caregivers with real-time feedback on their infants’ diets, along with current infant diet recommendations and a tracking form to assess how well they are complying with them [39]. The purpose of this study is to report on the effectiveness of this website among caregivers of infants in improving diet quality compared to the control arm, with the hypothesis that the intervention group will have improved diet outcomes.

## Methods

The Baby-Feed website trial was an individually randomized, parallel-group trial among 160 caregivers and their infants for 6 months to test if access to the intervention resulted in improvements in diet and weight (primary outcome) [39]. The results on weight outcomes are under review in another journal. The Baby-Feed website (www.babyfeed.org) is a secure website hosted on the Microsoft Azure cloud platform, providing access to the nutrition portal via a laptop or mobile phone. A detailed discussion of the development and pilot testing of the Baby-Feed web application has been published in prior studies [35, 36]. This online study follows the CONSORT 2025 reporting guidelines and the CONSORT checklist is provided in Additional File 1.The TIDieR Checklist for randomized clinical trials is provided in Additional File 2. Patients and the public were not involved in the design, conduct, and reporting of the trial. The Institutional Review Board (IRB) at Florida International University approved the study. The study was registered in August 2023 at ClinicalTrials.gov (NCT05990439). No changes were made to the original protocol after the commencement of the trial. Caregivers with eligible infants were provided with a full copy of the online IRB to read before signing the online informed consent to elect participation in the study.

### Recruitment

Participants were recruited from April to October 2024 with a combination of traditional (flyers, health care provider referrals), and online strategies (social media or university email blasts), as previously reported [39]. Upon starting recruitment, four participants were found to be fraudulent (i.e. did not have an infant); therefore, all further recruitment methods required a pre-screening survey (completed in Qualtrics) to assess eligibility and an additional phone call to validate eligibility. The verification phone call included specific questions, such as asking the caregiver to verify pediatrician name as well as upcoming infant well-baby visit dates that are typical in infant care. Those that could not answer, or provided an answer inconsistent with routine care, did not proceed to consent. Infant eligibility included: less than 4 months of age, term healthy infant at birth, no chronic illnesses or special diet. Caregiver eligibility included: be a primary caretaker living in the United States (US), speak and read in English or Spanish, have internet access via a laptop or mobile phone, own a smartphone with text capability, and be willing to participate for the full study duration of 6–9 months. Participants received incentives (electronic gift cards), once they enrolled and consented, and after completion of all tasks specific to the well-baby visit.

*Socio-demographics*: Caregivers also completed a baseline questionnaire via Qualtrics that included their relationship to the infant, birth year, race and ethnicity, highest level of education completed, number of children, Supplemental Nutrition Program for Women, Infants, and Children (WIC) participation, and self-reported weight and height. Their body mass index (BMI) as defined by WHO was calculated (kg/m^2^) and categorized as healthy, overweight, or obesity. Infant-specific questions included their baby’s gender, date of birth, birth weight, and length.

### Randomization

Caregivers were randomized in advance to either the intervention or control group using computer-generated block randomization for 160 participants, with an allocation ratio of 1:1. The randomization report was stored in a read-only Excel file on a secure university server, accessible by the primary investigator. The participants were not given awareness of their assignment; however, differing group tasks were defined in the IRB, enabling their group identity. The primary investigator provided each caregiver with a unique login and password to enter the Baby-Feed website from their phone or laptop, based on the group randomly assigned from the report. When completing the baseline questionnaires, caregivers provided the estimated date of their 4-month well-baby visit with their healthcare provider. Based on this date, reminder text messages were sent asking caregivers to access the website to complete the assessments directly on the website, to coincide with their well-baby visits, typically at 4, 6, and 9 months of age. These reminders were sent through Ripple Science, a website to monitor enrollment and assessment visits, allowing users to receive set automated texts and emails based on the scheduled visits. If tasks were not completed by expected dates, additional personalized texts and direct phone calls were made by the research team using Google Voice to verify dates and provide additional task reminders.

### Intervention group

The intervention group had access to the following components in the Baby-Feed website, which incorporated constructs from both the SCT [37] and HSET [38] in the development, as previously described [35]:

- *Infant Food Frequency Questionnaire* (FFQ): Two weeks before their estimated well-baby visit, caregivers were asked to complete a validated infant FFQ [40], which asks how many times the infant consumed certain foods and their serving size during the last 7 days. Upon completion, participants immediately received a screen pop-up with the results providing the total amounts of each food group consumed (milk, protein foods, whole and refined grains, fruits, vegetables, juices, sugary beverages, sweets, and salty snacks) and automated text with color feedback, of which were consumed “adequately, below, or above” and corresponding color coding as green, yellow and red, respectively. Participants were encouraged to discuss results with their healthcare provider at their upcoming well-baby visit.
- *Food Recommendations*: After each well-baby visit, the participant was asked to visit the “Food Recommendations” tab in Baby-Feed. The Food Recommendations section provides the translation of organizational guidance into more simplistic messaging in a few sentences and visual interpretations to provide education. The amounts recommended for each food group are based on the guidance from the American Academy of Pediatrics [6] , the World Health Organization (WHO) [4, 5] and the Dietary Guidelines for Americans for children 0-2 years [3]. These recommendations are specific to the age of the child, 0-6 months or 6-12 months, as the number of certain food groups changes over time and have been previously published [35]. For certain foods, such as juices, SSBs, sweets, and salty snacks, the recommended amount is zero. The participant is asked to view the recommendations and click submit when finished.
- *Food Tracking*: After each well-baby visit, participants were asked to complete a short form in the “Food Tracking” section on the website. It asked caregivers how well they complied with each food group recommendations, as described above, by clicking on a symbol, representing an “up arrow” (above), “down arrow” (below), or an “=” sign (equals). For example, salty snacks (0), and ideally will respond with a “=” sign, representing a 0 intake. Upon completion, it also provided automated text with color feedback of which was consumed “adequately, slightly below, or above” and corresponding color coding as green, yellow, and red, respectively.
- *Growth Charts*: After each well-baby visit, participants were asked to input the infant’s weight and length from the visit into this tab. Immediately, from the inputted data, the weight for length is calculated, plotted, and provided for view on the 0-24 months WHO growth chart. The feedback also includes a statement on whether the weight gain is adequate, above, or below, using the same text and color-coding system. If outside of the healthy growing range, the message prompted caregivers to discuss the results with their healthcare provider.
- *Educational Resources from Experts in Nutrition*: After each well-baby visit, the participant was asked to read or view 1 or more selections in this section, which was divided into 0-6 months and 6-12 months options. This section provided links to videos and newsletters from trusted nutrition organizations to complement the information provided in Baby-Feed.

### Control group

Caregivers in the control group were asked to complete the same infant FFQ and to input the weight and length in the “Growth Chart” tab at the same time points, before and after their well-baby visit at ~ 4, 6, and ~ 9-months in the Baby-Feed website, but they did not receive instantaneous growth feedback, nor were able to access the Food Recommendations, Food Tracking, or Educational Resources sections. They did not receive any alternative set of resources.

### Outcomes

The research team was not masked as all assessments were self-reported by participants, as described below.

*Diet quality*: this was analyzed from the self-reported responses to the infant FFQ for both groups directly on the website. The website automatically calculates the intake of each food group (milk, whole grains, refined grains, proteins, vegetables, fruits, 100% juices, sugar-sweetened drinks, sweets, and salty snacks) and the Diet Quality Index Score (DQIS), as previously described [41]. Briefly, this score is automatically calculated as follows: for infants 0-5.9 months, the milk is scored as 15 points if breastfeeding exclusively, 10 points if breastfeeding partially, and 5 points if formula feeding only; for the other food groups, 5 points is given if the food group has not been introduced, and 0 points if it was introduced. For 6–12 month intake of milk/formula and solid foods, 5 points were assigned for appropriate intake, 2.5 points if the intake was slightly above or below, and 0 points if the intake was above or below recommendations. For grains, the score was equally divided between refined and whole grains, to sum 5 points if appropriate intake for both. For milk, 15 points were assigned for breastfeeding exclusively, 10 points if breastfeeding partially, and 5 points if formula feeding within the recommendations. The total DQIS and individual food groups’ scores were calculated overall and by sex at baseline and at the end of the study. The DQIS was also categorized as excellent (score = 45–55); good (score = 35–44); needs improvement (score = 25–34); and poor (score = 0–24). These categories were utilized, as this scoring system was developed for the DQIS [40].

### Compliance with the intervention

Compliance and adherence with the intervention was monitored frequently by the research team, sending multiple text messages and calls to remind participants to complete the study tasks. Only when tasks were completed did the research team manually record the corresponding date onto a monitoring spreadsheet, the e-gift was sent, and the caregiver continued with the study.

### Statistical analyses

*Sample Size*: The target sample size was calculated based on the rate of weight gain from a previous pilot trial [35]. We used infant weight because previous interventions have more consistently demonstrated effects on dietary outcomes than on anthropometric measures. Given that changes in weight are typically smaller and more difficult to detect, we powered the study to ensure adequate sensitivity for this outcome. Consequently, the study is also sufficiently powered to detect changes in dietary outcomes, which generally require smaller sample sizes. A total of 67 participants per group would be necessary to detect a similar effect size, with an additional 20% participants added to allow for dropouts, resulting in a targeted sample of 80 participants per group.

*Data Analysis*: For descriptive analyses, means and standard deviations were used for continuous variables. Differences in socio-demographic variables by groups (intervention vs. controls) were analyzed using independent sample t-tests for continuous variables and Chi-square for categorical variables. To compare overall DQIS scores and individual food groups’ scores from baseline to the end of the study between groups and by sex, analysis of covariance was used, adjusting for infant age at baseline and end of the study. A paired t-test was used to analyze changes over time within each group. The differences in the categorized DQIS score at the end of the study were analyzed using Chi-square, overall, and stratified by infant sex. As the majority of DQIS were in the excellent and good categories, this variable was re-categorized as the median or above (45) or below. There was no missing data for the participants analyzed. An infant in the control group found to have faltering weight was flagged as a possible data entry error. In a telephone discussion with the caregiver, the infant was found to have an extended illness and consequently, surgery, and was removed from the analysis.

## Results

**Fig. 1:**
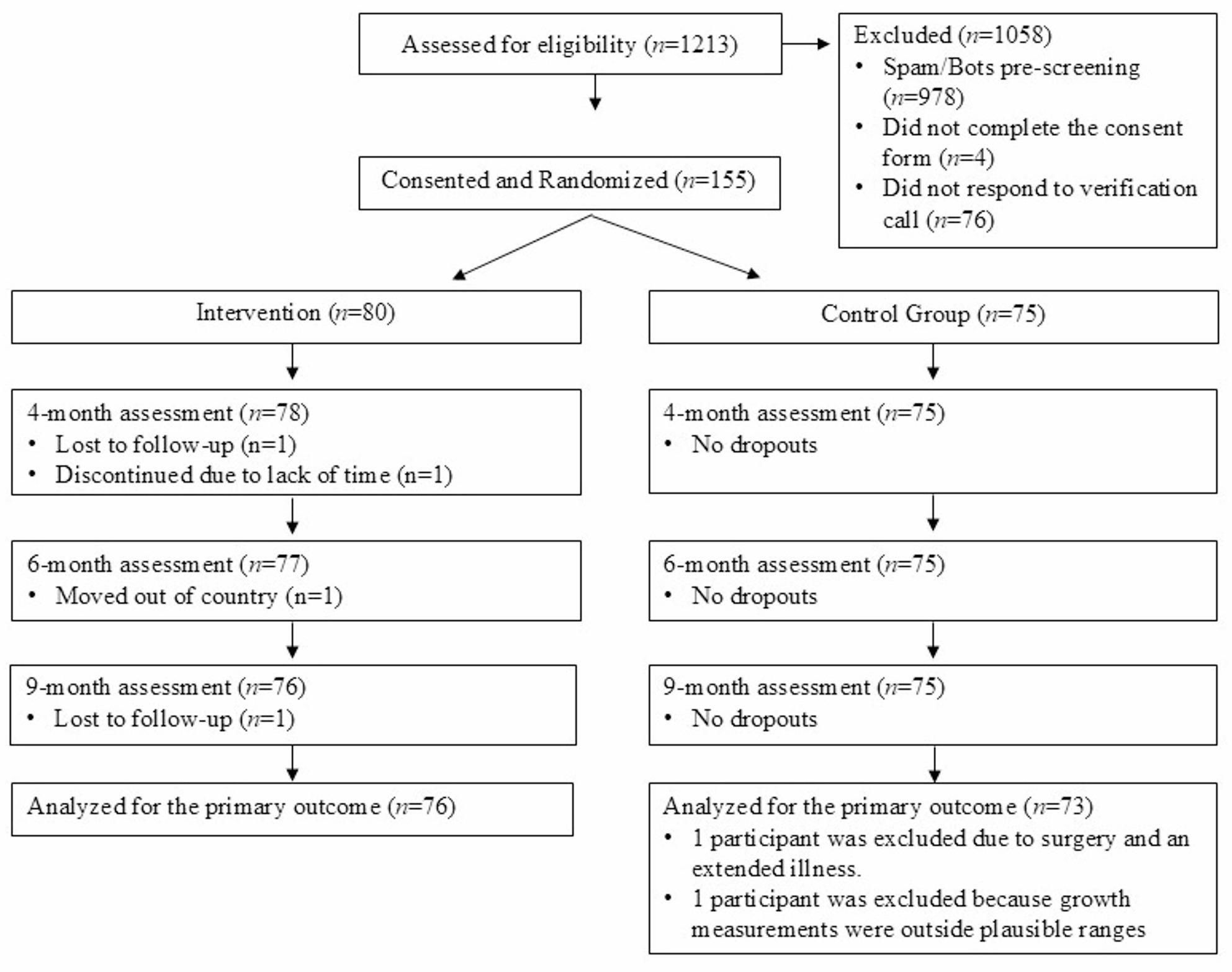
CONSORT 2025 Diagram of Participants

A total of 155 caregivers were enrolled and randomized into the intervention group (*n* = 80) or the control group (*n* = 75) (Fig. 1). Most completed the trial (76 in the intervention and 75 in the control) with the last participant completed in August 2025. No harm or unintended event was identified in either group.

Baseline characteristics of the caregivers are reported in Table 1, with no statistical differences between groups. Overall, the caregiver’s mean age was 34.2 years, 24.5% identified as Hispanic, 65.2% identified as White, 47.1% had a graduate degree, 52.3% had 1 child, 16.1% were WIC participants, 43.9% were overweight, and 23.9% had obesity.

**Table 1:** Socio-demographic characteristics of caregivers and infants

Characteristic	Intervention Group *N* = 80 *N* (%) or Mean ± SD	Control Group *N* = 75 *N* (%) or Mean ± SD	*P*-value
Caregivers			
Age (years)	34.3 ± 5.14	34.1 ± 4.89	0.749
Sex			1.00
Females	76 (95)	72 (96)	
Males	4 (5)	3 (4)	
Race			0.390
Asian/Pacific Islander	9 (11.3)	3 (4)	
African American/Black	5 (6.3)	6 (8)	
Caucasian/White	50 (62.5)	51 (68)	
Mixed Race	1 (1.3)	3 (4)	
Not reported	15 (18.8)	12 (16)	
Ethnicity, Hispanic	17 (21.3)	21 (28)	0.329
Level of education			0.918
High School/GED	5 (6.30)	5 (6.70)	
Associate’s/Trade	7 (8.80)	9 (12.0)	
Bachelor’s	29 (36.3)	27 (36.0)	
Graduate (MS, PhD, MD/DO)	39 (48.8)	34 (45.3)	
Number of Children			0.343
1	44 (55.0)	37 (49.3)	
2	30 (37.5)	26 (34.7)	
3 or more	6 (7.50)	12 (16.0)	
WIC Participant	9 (11.3)	16 (21.3)	0.068
Caregiver BMI			0.671
Healthy Status (18.5–24.9)	28 (35.0)	22 (29.3)	
With Overweight Status (25.0- 29.9)	35 (43.8)	33 (44.0)	
With Obesity Status (> 30.0)	17 (21.3)	20 (26.7)	
*Infants*			
Sex			0.521
Females	41 (51.2)	34 (45.3)	
Males	39 (48.8)	41 (54.7)	
Birth weight (kg)	3.39 ± 0.582	3.24 ± 0.531	0.097
Birth length (cm)	50.5 ± 3.09	51.1 ± 2.68	0.206

The Infant DQIS component and total scores by groups are shown in Table 2. At baseline, there were no differences in the total DQIS or in the individual food groups’ scores, in which most infants were primarily drinking breastmilk or infant formula or both. At the completion of the intervention, Infant DQIS was significantly higher in the intervention group (44.6 ± 6.53) compared to the control group (42.5 ± 7.5), after adjusting for infant’s age and energy intake (*p* = 0.046). No outliers were found. When looking at the individual components, we also found that infants in the intervention group had significantly higher scores for whole grains, proteins, and salty snacks but lower scores for fruits compared to infants in the control group, in the adjusted models (*p* < 0.05). Also, infants in the intervention group showed a trend for higher scores in 100% juices, SSB, and sweet foods (p-values 0.056–0.061). When stratified by sex, Infant DQIS was higher in males in the intervention group (44.8 ± 6.87) compared to the control group (41.8 ± 7.36) in the unadjusted model (*p* = 0.037), but after adjusting for age and energy intake, there was a trend only (*p* = 0.061). Differences in the infant DQIS between groups were observed in females at the baseline visit (*p* = 0.003) in the adjusted model, but not at 9 months (*p* = 0.368). To explore what could explain the sex differences in the study, we compared the socio-demographics by the infant’s sex and found no significant differences (data not shown). There were also significant differences in the individual food groups at 9 months, with higher scores in the intervention group for salty foods (less consumption).

**Table 2:** Total Infant Diet Quality Score (Infant DQIS) and individual food groups score at baseline and at the end of the study

Score	Baseline (4 months)						End of study (9 months)					
	Control		Intervention		Differences between groups		Control		Intervention		Differences between groups	
	Mean	SD	Mean	SD	Unadjusted *p*-value	Adjusted*p*-value^1^	Mean	SD	Mean	SD	Unadjusted *p*-value	Adjusted *p*-value^1^
Overall	*N*=73		*N*=76				*N*=73		*N*=76			
Total DQIS	52.6	4.31	53.0	4.33	0.282	0.400	42.5	7.55	44.6	6.53	**0.036**	**0.046**
Milk	13.0	4.05	13.3	3.79	0.311	0.257	9.46	5.69	10.5	5.06	0.128	0.288
Grains												
* Whole* ^2^	2.40	0.50	2.50	-	0.039	0.488	1.22	1.27	1.22	1.17	0.498	**0.042**
* Refined*^2^	2.45	0.32	2.45	0.32	0.485	0.383	2.26	0.89	2.21	0.78	0.331	0.054
Protein	4.97	0.29	4.95	0.43	0.398	0.476	3.92	1.90	3.92	1.80	0.494	**0.030**
Vegetables	5.00	0.00	4.93	0.57	0.163	0.435	3.07	1.83	3.62	1.60	**0.028**	0.980
Fruit	4.93	0.58	4.90	0.64	0.378	0.509	3.85	1.61	3.68	1.61	0.263	**<0.001**
100% juice	4.93	0.58	5.00	0.00	0.156	0.705	4.66	0.96	4.77	0.73	0.219	0.056
SSBs	4.97	0.29	5.00	0.00	0.156	0.639	4.83	0.76	4.93	0.40	0.148	0.061
Sweet Foods	5.00	-	5.00	-	-	0.528	4.83	0.63	4.93	0.40	0.117	0.057
Salty Foods	5.00	-	5.00	-	-	0.528	4.66	0.96	4.80	0.68	0.150	**0.045**

Females	*N*=35		*N*=41				*N*=35		*N*=41			
Total DQIS	54.0	2.92	53.3	3.81	0.199	0.003	43.4	7.77	44.5	6.30	0.249	0.368
Milk	14.1	2.84	13.3	3.81	0.140	0.287	10.7	5.24	10.5	5.01	0.424	0.245
Grains												
* Whole*^2^	2.50	-	2.50	-	-	0.003	0.823	1.32	1.04	1.21	0.229	0.395
* Refined*^2^	2.39	0.47	2.50	-	0.073	0.812	2.36	0.79	2.29	0.83	0.368	**0.002**
Protein	4.93	0.42	5.00	-	0.141	0.846	3.86	1.95	3.78	1.86	0.431	0.430
Vegetables	5.00	-	5.00	-	-	0.956	3.21	1.78	3.71	1.50	0.094	0.538
Fruit	5.00	-	5.00	-	-	0.956	3.93	1.75	3.78	1.49	0.346	**0.044**
100% juice	5.00	-	5.00	-	-	0.956	4.57	1.13	4.76	0.75	0.199	0.454
SSBs	5.00	-	5.00	-	-	0.956	4.71	1.01	4.94	0.39	0.096	0.595
Sweet Foods	5.00	-	5.00	-	-	0.956	4.79	0.71	4.88	0.54	0.262	0.393
Salty Foods	5.00	-	5.00	-	-	0.956	4.64	1.07	4.88	0.55	0.111	0.394

Males	*N*=39		*N*=35				*N*=39		*N*=35			
Total DQIS	51.4	4.99	52.7	4.91	0.131	0.429	41.8	7.36	44.8	6.87	**0.037**	0.061
Milk	11.9	4.68	13.3	3.82	0.089	0.217	8.33	5.91	10.4	5.20	0.056	0.641
Grains												
* Whole*^2^	2.31	0.67	2.50	-	0.048	0.552	1.57	1.13	1.43	1.10	0.291	**0.042**
* Refined*^2^	2.50	-	2.39	0.47	0.073	0.053	2.18	0.74	2.11	0.73	0.337	0.062
Protein	5.00	-	4.89	0.63	0.147	0.071	3.97	1.88	4.07	1.72	0.409	**0.033**
Vegetables	5.00	-	4.86	0.85	0.147	0.389	2.95	1.89	3.50	1.74	0.099	0.102
Fruit	4.87	0.80	4.79	0.93	0.335	0.422	3.78	1.50	3.57	1.75	0.289	**0.009**
100% juice	4.87	0.80	5.00	-	0.173	0.752	4.74	0.77	4.79	0.71	0.404	0.061
SSBs	4.94	0.40	5.00	-	0.173	0.599	4.94	0.40	4.93	0.42	0.470	0.070
Sweet Foods	5.00	-	5.00	-	-	0.515	4.87	0.56	5.00	-	0.090	0.084
Salty Foods	5.00	-	5.00	-	-	0.515	4.68	0.85	4.71	0.81	0.429	0.061

^1^ANCOVA adjusted for age and energy intake at each visit. ^2^The score for grains was divided equally between refined (2.5 points) and whole grains (2.5 points). Values with (-) are shown when there was not computed standard deviation due to all the observations having the same value. Significant differences between groups (p<0.05) are in bold. Total DQIS and the individual food groups’ scores significantly decreased over time within each group (*p*<0.05), except for the intervention group for sweet foods and for control and intervention for SSBs [p-values not shown in table]

Table 3 shows the logistic regression for the Infant DQIS categorized. The logistical regression model showed that infants in the control group had twice the odds of having an infant DQIS below the median score of 45 (OR = 2.00; 95% CI: 1.012–3.950; *p* = 0.046) compared to the intervention group, after adjusting for age and energy intake. When stratified by sex, the significance was lost.

**Table 3:** Logistic regression for DQIS below the median at 9 months by group and sex

Group	DQIS < 45 (median) at 9 months					
	Unadjusted			Adjusted^1^		
Overall	OR	95% CI	*P*-value	OR	95% CI	*P*-value
Control	1.760	0.919, 3.368	0.088	2.00	1.012, 3.950	0.046
Intervention	1			1		
Males						
Control	2.044	0.804, 5.199	0.133	2.306	0.875, 6.078	0.091
Intervention		1			1	
Females						
Control	1.476	0.592, 3.676	0.403	1.674	0.622, 4.506	0.308
Intervention		1			1	

^1^ Adjusted for infant age and energy intake

We sought to analyze whether a greater engagement with the Baby-Feed website (number of times caregivers completed the Food Tracking, Growth Chart, read the Food Recommendations, and accessed the additional Educational Resources) was correlated with better results, but because of technical issues with the Baby-Feed website, this was not properly recorded.

## Discussion

The present trial found that access to the Baby-Feed intervention resulted in a significantly higher infant DQIS in the intervention group compared to the control group. Although only a small difference was found in the total DQIS (2 points) between groups, this reflects meaningful changes in overall diet quality beyond this 2-point difference, as this score is based on multiple components of the diet (milk, whole grains, refined grains, proteins, vegetables, fruits, 100% juices, sugar-sweetened drinks, sweets, and salty snacks). In fact, we found significant improvements in the individual scores for whole grains, fruits, and salty snacks compared to the control group and trends for a better score for 100% juices, SSB, and sweet foods. This suggests a diet quality that is more closely compliant with the current dietary recommendations for these food groups. These results appeared to be driven mainly by male infants. Also, there was a trend toward a greater proportion of infants in the intervention group with a total Infant DQIS score categorized as ‘excellent’ than in the control group. The focus of Baby-Feed on real-time feedback after assessing the infant’s diet aligns with preventive care and today’s expectations of instantaneous answers, given that web-based platforms are easily accessible and a preferred source of parenting information.

Other technology-based interventions have also been successful in improving overall diet quality and infant feeding practices [28–30, 42]. For example, the Early Food for Future trial tested an eHealth intervention for 718 parents with access to monthly website videos on specific feeding topics and baby food recipes for ages 6–12 months, compared with standard care [28]. At 12 months, infants in the intervention group consumed more fruit and vegetables (*p* = 0.035), and more vegetables were offered/tasted (*p* = 0.015). In the Grow2Gether trial with 87 mothers, the intervention group participated in online peer groups of 10–14 mothers, and posted curriculum facilitated by a psychologist [29, 30]. At the end of the study, mothers in the intervention group were less likely to feed their infant cereal in a bottle to their infant. These interventions showcase that integrating dietary guidance, by healthcare provider and/or using technology, can result in improved infant diet quality and adherence to dietary guidelines by infant caregivers.

It was interesting to note sex differences in the results of the present study, whereas infant males in the intervention seem to have a higher diet quality and a higher proportion classified in the highest diet quality group compared to the control group, while this was not observed the female infants. This may represent a higher likelihood of having stricter adherence to infant diet recommendations among caregivers of male infants. As part of the INSIGHT trial, sex differences in maternal restrictive feeding practices were examined [43]. Mothers in the intervention group reported greater feeding restriction by limiting food quantity for male infants at 28 weeks (*p* = 0.07), and at their annual well-child visits at 1, 2, and 3 years (*p* = 0.04). Another study with 90 Latino mother-infant dyads showed that girls were more likely to be continued on breastmilk than boys [44]. Although published research on the sex differences in infant feeding remains limited, this publication provides additional evidence. However, more research on the impact of cultural values and gender-based beliefs on feeding practices between infant sexes is warranted to understand the differences in obesity risk.

It is also important to note that diet quality was high (in the excellent range) at 4 months before the intervention started, reflecting that most participants were following the general infant feeding recommendations of mainly feeding milk (breast or formula). Only a few caregivers had introduced solid foods, and even fewer juice or SSBs as the scores were close to 5, which indicated that they had not introduced any other food other than milk. At the end of study, the score for most food groups significantly dropped compared to baseline in both groups, although the score for 100% juice, SSBs, salty foods, and sweet foods scores were somewhat close to 5, meaning they were following the general guidance to avoid these foods in the 1st year. A longer follow-up is needed to explore if these scores continue to decrease in the toddler years.

The study had several limitations and strengths that are worth noting. Among the limitations, although the recruitment was online to allow a diverse group to enroll, some groups remained underrepresented. The education level was high, with many participants having a professional and/or doctorate-level, which may have impacted the results, as diet quality has been shown to be higher among parents/caregivers of higher educational level [44, 45]. This could have contributed to a ceiling effect and consequently less opportunity for improvement in response to the intervention, although small (yet significant) improvements in total DQIS and in some individual components were seen. This higher educational level could be explained by the snowball recruitment from online community groups that catered to healthcare professionals. As all data was self-reported, human error may have occurred with data input into the website, as well as measurement error of the growth measurements obtained at the well-baby visit. FFQs may not be as accurate as other quantitative diet assessment tools because they rely on a predetermined food list and assumptions about culturally specific foods, although the FFQ used was validated for infants [40]. Infants often have small portions when trying new foods, which may not be easily accounted for, even with visual portion-size examples and multiple serving-size options.

It was not possible to determine which of the digital components in Baby-Feed (food results, growth chart results, food recommendations, food tracking results, and additional educational resources) contributed the most to the overall intervention effectiveness. Although caregivers were encouraged to return to the Baby-Feed website as often as desired, there were technological issues with the recording of the number of times that participants accessed these digital component tabs and how long they engaged on the platform each time they logged in. Some participants reported emails, text messages, and phone calls going directly to their junk/spam folders. Lastly, the trial was not masked, however, all assessments were self-reported directly in the webpage and the diet was automatically calculated in the webpage, reducing the bias from the lack of masking.

Among the strengths, this study had a very high retention and adherence. As seen in other studies with caregivers of infants [34, 46–49], this is a highly motivated group with generally high retention rates. Also, the tasks were relatively easy, with a reasonable expectation of time dedicated to the study. The study was completely online, with visits coinciding with the well-child visits for the recording of weight and length, which facilitated the completion of assessments. Participants also received financial compensation after each assessment. The intervention was developed using trusted theories and this assisted with the facilitation of feeding practices to strengthen self-efficacy of caregivers, as previously published [49]. Lastly, automated and personalized text messages also assisted with high retention and adherence. Future studies should consider pairing the Baby-Feed intervention with the healthcare providers, so that caregivers receive the same messages from different sources. To obtain continued behavior change, the website could be combined with personalized text messages that link back to the website for increased, more regular engagement, as well as becoming a standardized resource that the infant health care provider discusses and reinforces at well-baby visits and beyond. Also, the Baby-Feed webpage requires a few technical improvements to be more user-friendly and intuitive. As participants found the additional educational resources valuable [49], future studies could establish additional collaborations with trusted organizations to give participants access to other educational resources.

## Conclusions

The Baby-Feed website led to improvements in the diet quality in infants, particularly in male infants. Since caregivers are primarily responsible for establishing healthy eating patterns, and the rates of childhood obesity are increasing in the US, more accessible tools and resources like Baby-Feed are needed to help caregivers receive automatic feedback on how well they are feeding their infants. Also, this relatively easy and accessible website could be a valuable resource to complement the nutrition care provided by healthcare providers to ensure caregivers receive consistent and valid information on infant feeding practices that can increase their confidence to adopt a healthy diet quality early in life.

## Supplementary Information

Additional File 1: CONSORT Checklist for Randomized Controlled Trial.

Additional File 2: TiDieR Checklist.

## Data Availability

The datasets used/analyzed during the current study are available from the current author upon reasonable request.

## Abbreviations

CFBs: Complementary foods and beverages
SSBs: Sugar-sweetened beverages
US: United States
SCT: Social Cognitive Theory
HSET: Health Self-Empowerment Theory
IRB: Institutional Review Board
FFQ: Food Frequency Questionnaire
WHO: The World Health Organization
DQIS: Diet Quality Index Score
WIC: Supplemental Nutrition Program for Women, Infants, and Children
BMI: Body Mass Index
